# Terahertz Metamaterials for Biosensing Applications: A Review

**DOI:** 10.3390/bios14010003

**Published:** 2023-12-21

**Authors:** Wu Zhang, Jiahan Lin, Zhengxin Yuan, Yanxiao Lin, Wenli Shang, Lip Ket Chin, Meng Zhang

**Affiliations:** 1 School of Physics and Material Science, Guangzhou University, Guangzhou 510006, China; zhangwu@gzhu.edu.cn (W.Z.); 2112119048@e.gzhu.edu.cn (J.L.); yuanzhengxin@gzdx.wecom.work (Z.Y.); 2112319064@e.gzhu.edu.cn (Y.L.); 2 School of Electronics and Communication Engineering, Guangzhou University, Guangzhou 510006, China; shangwl@gzhu.edu.cn; 3 Key Laboratory of On-Chip Communication and Sensor Chip of Guangdong Higher Education Institutes, Guangzhou 510006, China; 4 Department of Electrical Engineering, City University of Hong Kong, Kowloon, Hong Kong SAR 999077, China

**Keywords:** biosensing, terahertz, metamaterials, resonance

## Abstract

In recent decades, THz metamaterials have emerged as a promising technology for biosensing by extracting useful information (composition, structure and dynamics) of biological samples from the interaction between the THz wave and the biological samples. Advantages of biosensing with THz metamaterials include label-free and non-invasive detection with high sensitivity. In this review, we first summarize different THz sensing principles modulated by the metamaterial for bio-analyte detection. Then, we compare various resonance modes induced in the THz range for biosensing enhancement. In addition, non-conventional materials used in the THz metamaterial to improve the biosensing performance are evaluated. We categorize and review different types of bio-analyte detection using THz metamaterials. Finally, we discuss the future perspective of THz metamaterial in biosensing.

## 1. Introduction

Nowadays, biosensing has become a more and more import research topic in the fields of human health care, disease diagnosis and drug development, etc. Various sensing mechanisms such as mechanical detection and electrical sensing have been studied for a long period [1]. In mechanical biosensing, different mechanical resonators including microcantilevers [2], micropillars [3] and piezoelectric structures [4] were proposed, which usually require complicated fabrication processes. In electrical biosensing, the bio-analytes can be detected by observing the impedance change [5], dielectrophoresis effect [6] and signal change in field effect transistors [7]. In the above detection mechanisms, the analytes have to be attached to the mechanical resonator or electrical circuit to change the detecting signal, which will inevitably harm the bio-analytes. A non-destructive detection mechanism is then preferred. Terahertz (THz) waves, which lie between microwave and infrared frequencies, can interact with various biological samples, such as proteins, DNA and cells, through their specific vibrational modes. By analyzing the signal change when the THz wave interacts with these biological specimens, valuable information about their composition, structure and dynamics can be extracted. As THz waves have lower energy compared to X-rays or gamma rays, THz-based biosensing is safe for both the sample and the operator. In addition, THz waves are non-destructive, allowing repeated measurements without damaging the biological sample. Therefore, THz wave technology has emerged as a promising approach for biosensing applications like disease diagnosis, drug discovery and food quality assessment. However, the wavelength of THz waves ranges from 30 µm to 3000 µm, which is much larger than most bio-specimens such as viruses, bacteria and some other biomolecules. This significantly limits the detection sensitivity of THz-based biosensing. To improve the biosensing performance, one promising approach is to enhance the THz wave resonance in the micro- or nano-sized bio-specimens, which can be realized through the design of metamaterials.

Metamaterials are artificial materials that manipulate electromagnetic (EM) waves through their array of meta-atoms or unit cells [8,9,10]. The manipulated EM wave ranges from the microwave to the visible-light regime by simply designing the corresponding unit cells on a subwavelength scale. Metamaterials are thus fabricated through different processes such as printed circuit board (PCB) technology, optical lithography and electron beam lithography (EBL) for microwave [11], THz wave [12] and visible-light control [13], respectively. Different unit cell structures, such as the split-ring resonator [14,15,16], the fishnet [17,18] and the gammadion shape [19], are designed to induce different resonances of the EM wave. As a result, various novel properties of the EM wave, including negative refraction [20,21], perfect absorption [22,23], strong optical activity [24,25,26] and extraordinary transmission [27,28,29], can be realized, which provide a fascinating platform to realize broad applications such as invisible cloaking [30,31,32], superlenses [33,34,35] and polarization conversion [36,37,38]. In addition, two-dimensional metamaterials, or metasurfaces, have received intensive research attention recently, which control the wavefront of the EM wave and realize functions including flat-lens focusing [39,40], beam shaping [41] and imaging [42,43,44], etc. The resonance of metamaterials is also affected by the environmental property surrounding the metamaterials. A small change in the surrounding property could induce a significant difference in the resonant frequency or amplitude of the EM wave; the phase and the polarization of the EM wave may also be tuned. Therefore, metamaterials are commonly designed as refractometers that detect refractive-index change.

THz metamaterial technology, which manifests the advantages of both non-destructive sensing and high sensitivity, is an attractive platform for biosensing applications. There have been several review papers on THz metamaterials for biosensing [45,46,47]; for example, a recent review on THz metamaterial biosensing focused on the structure design of the metamaterials [47]. In this review, we aim to provide a deeper and more comprehensive discussion on THz metamaterial biosensing, including different signals of the THz wave used for detection, various resonances induced in the metamaterials and novel materials used for the metamaterials, to improve the biosensing performance. We also categorized different types of analytes detected through the THz metamaterials. This review work will provide a better understanding of the THz metamaterial design and working principle for biosensing and guidance for the community to innovate a sensitive THz metamaterial for different analytes.

The structure of the review paper is as follows: We first discuss different THz wave sensing principles employed in THz-metamaterial-based biosensors in Section 2, which include transmission peak shift, absorption peak shift, polarization rotation and THz imaging. Then, we categorize different THz metamaterial resonances for signal enhancement, such as electromagnetically induced transparency (EIT)-like resonance, Fano resonance, toroidal resonance, surface plasmon polariton (SPP) resonance and resonance related to bound states in the continuum (BIC) in Section 3. Section 4 introduces the THz metamaterials based on non-conventional materials to improve the biosensing performance, which include all-dielectric materials, carbon materials, nanoparticles (NPs) and nanowires (NWs). Finally, the detection of different analytes, such as chemical agents, biomolecules, microorganisms and cells, is investigated in Section 5. The outlook and conclusions of THz metamaterials for biosensing are discussed in Section 6.

## 2. THz Wave Sensing Principles

The amplitude, phase and polarization of the THz wave could be varied when the wave is incident on the metamaterials surrounded, coated or bound with different types or concentrations of analytes. Therefore, detecting a signal change in the THz wave could be applied for biosensing. This section will discuss the biosensing principles in different THz metamaterials, including resonant frequency shift, wave polarization conversion or rotation and transmission imaging.

### 2.1. Transmission Dip Shift

**Figure 1:**
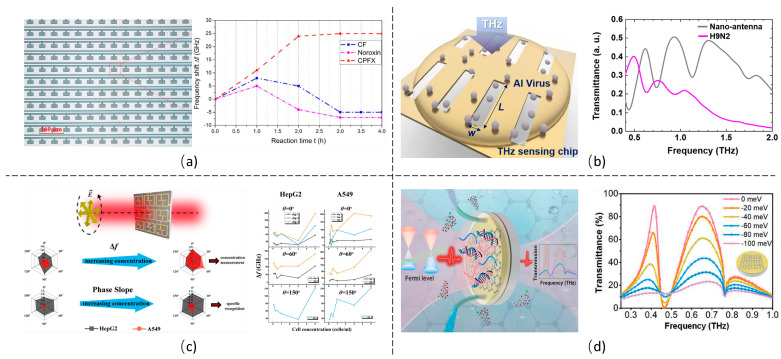
Biosensing based on transmission and absorption peak shift of THz waves through metamaterials. (**a**) Transmission dip shift metamaterial for BSA detection [50]; (**b**) metamaterial transmission spectrum with multi-resonance [51]; (**c**) polarization-dependent transmission shift metamaterial [53]; (**d**) transmission-amplitude-change-based metamaterial [54].

Metamaterials manifest strong resonance at a specific frequency depending on the metamaterial structure and ambient refractive index, usually inducing a dip in the transmission spectrum. The transmission dip shifts when the metamaterial is coated or bound with a biosample, which is more significant with higher concentration. For example, Wang et al. proposed a metamaterial with a split-ring resonator unit cell structure and observed a polarization-insensitive transmission frequency shift from 1.46 THz to 1.19 THz after the metamaterial was bound with 3 mmol/L bovine serum albumin (BSA) solution [48]. This led to a sensitivity of 95 GHz/(mmol/L) and a detection resolution of 17.7 µmol/L for BSA. The metamaterial transmission dip shift can also be applied to differentiate similar types of chemicals. For example, a larger transmission dip shift was observed from the THz metamaterial coated with aflatoxin B1 than that with aflatoxin B2, even though the two chemicals were at the same concentration [49]. In addition, the metamaterial transmission dip shift can be used to monitor chemical reaction processes. Hu et al. investigated a real-time interaction between the BSA solution and four different drug solutions through a THz metamaterial with micropillar unit cells, as shown in Figure 1a [50]. Transmission dips were induced at four different frequencies, respectively, which all induced a blue shift in the first hour of the reaction time and a red shift afterward. The resonant frequencies became stable after two hours, indicating the reaction was completed. For optimizing the sensitivity, metamaterials with multi-band transmission resonance [51] (Figure 1b) and high-plasmon modes were studied [52]. It was found that a higher sensitivity can be obtained from the shift of the higher-ordered resonant mode. For example, a metamaterial with three transmission dips at 0.71 THz, 0.24 THz and 0.31 THz was reported to have the sensitivity of 408 GHz/RIU (RIU: refractive-index unit), 552 GHz/RIU and 724 GHz/RIU, respectively, among which the highest-ordered resonant mode had the highest sensitivity [52]. The detection sensitivity can also be improved by setting the incident THz wave at an optimized polarization condition. This was achieved by designing an anisotropic metamaterial (Figure 1c) and plotting a radar map for its transmission dip shift at different polarization states. By choosing a proper polarization condition for the incident wave, larger transmission resonant shifts up to 213 GHz and 198 GHz were obtained for A549 cells and HepG2 cells at the concentration of 5 × 10^5^ cells/mL [53].

### 2.2. Absorption Peak Shift

A strong absorption peak can be observed in a well-designed metamaterial absorber, consisting of unit cell structures on the top and a reflected metallic layer on the bottom of the substrate. The absorption *A* of the metamaterial is obtained by 1−R, where *R* is the measured reflectance, assuming there is no transmission through the metamaterial. The absorption peak frequency mainly relies on the metamaterial impedance, matching with the environment. Therefore, bio-analytes on the metamaterial will induce a resonance shift in the absorption resonance frequency. For example, a loop-shape-based metamaterial absorber, which had an initial absorption peak at about 1.5 THz, caused a peak frequency shift after being surrounded with organochlorine pesticide residues and recorded a detection sensitivity of 0.213 mg/L [55]. A high sensing linearity with a regression coefficient of 0.9863 was achieved. In addition, the sensitivity can be improved by increasing the contact area between the bio-analytes and the metamaterial. Zhou et al. proposed a bi-layered metamaterial structure on which more analytes can be bound on both layers than single-layered metamaterials [56]. As a result, the contact area between the analyte and the metamaterial increased. Furthermore, the space between the two layers of the metamaterial manifested a hot spot under THz wave incidence. Therefore, a slight refractive-index change of the analyte will lead to a significant drift in the electrical field, resulting in a large shift in the absorption resonance. A sensitivity of 153 GHz/µM was experimentally demonstrated for biotin detection, which reached 10-fold higher than conventional cross-shaped metamaterials. A similar hot spot was observed in the microfluidic channel between the metamaterial resonant and reflected layers [57]. In the microfluidic channel, even a minute amount of analytes is sufficient for detection, which has the advantage of detecting high-cost and precious samples. When the analyte is injected into the microchannel, the refractive index of the space between the resonant element and the reflected layer will change; the hot spot is largely affected and, therefore, changes the resonant frequency. A sensitivity of approximately 0.2 THz/RIU was experimentally demonstrated by injecting air ($n_{\mathrm{air}}=1.0$), ethanol ($n_{\mathrm{ethanol}}=1.6$) and glucose ($n_{\mathrm{glucose}}=2.1$) into the microchannel, respectively. In addition, a multi-band THz metamaterial absorber was proposed to improve the sensing performance. The unit cell of such metamaterial is usually designed as a hybrid structure, which provides different resonant paths. One proper resonance is then selected to optimize the bio-detection sensitivity. For example, through a multi-band absorber consisting of unit cells with a hybrid structure of a square and a split-ring resonator, 2,4-dichlorophenoxyacetic acid was detected with a detection limitation as low as 0.1 pp and a regression coefficient above 0.95 [58]. Some bi-functional metamaterial that senses both the refractive index and the temperature of bio-analytes was later explored through metamaterial absorption. Besides the transmission dip and absorption peak shifts, other amplitude-dependent sensing principles such as transmission [54] (Figure 1d) or reflection amplitude changes [59] are also used for biosensing applications. The change of the metamaterial reflection amplitude was reported to enhance the sensitivity of the attenuated total reflection sensor, and the sensitivity was found to be improved fourfold [59].

### 2.3. Polarization and Chirality

**Figure 2:**
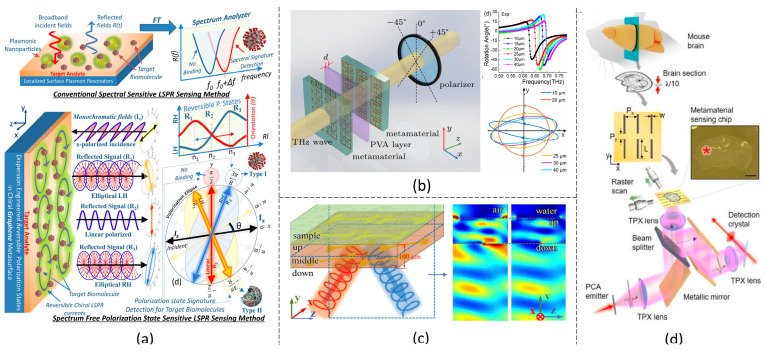
Biosensing based on polarization or imaging detection. (**a**) A chiral THz metamaterial for polarization-selective virus sensing [62]; (**b**) a chiral THz metamaterial for biosensing based on polarization conversion [63]; (**c**) a dual-layer chiral metamaterial for protein denaturation sensing based on THz circular polarization [64]; (**d**) brain-tissue imaging through terahertz metamaterial [67].

Besides resonance shift, the coating or binding of bio-analytes on the metamaterials can also induce a polarization conversion or rotation of the THz wave transmitted through the metamaterials. Liu et al. demonstrated a cell proliferation sensing metamaterial with a hexagon structure, through which the polarization angle of the THz wave rotated from −39° to 4°, and the ellipticity changed from −15.5° to 26° when detecting tumor cells [60]. The detection limit based on the polarization rotation reached 3.0 × 10^3^ cells/mL, which was 10-fold better than resonance shift sensing using the same metamaterial. Another metamaterial based on chirality is intrinsically suitable for biosensing through the detection of THz wave polarization change [61,62,63,64], as shown in Figure 2a–c. The chiral material has a unique unit cell structure that does not overlap with its mirror-image structure. There is a direct interaction between the electrical and magnetic responses in the chiral material, which causes different effective refractive indices for the left circularly polarized light and the right circularly polarized light. As a result, the polarization direction will be rotated, resulting in a polarization rotation phenomenon called optical activity. The optical activity is insignificant in nature chiral material, such as L-amino acids, D-sugars and nuclear acid. The chiral metamaterial is then proposed, which improves the optical activity for several orders [19]. When depositing bio-analytes on the chiral metamaterial, the elliptical angle and the circular dichroism of the THz wave change. Moreover, polarization sensing can differentiate analytes of different chirality types, which is usually not achievable through the detection based on resonance shift [65]. The polarization-based biosensing method only requires polarization measurement at a single frequency and does not require a broadband frequency scan as resonant shift sensing does. Therefore, polarization sensing can further simplify the experimental setup.

### 2.4. Transmission Imaging

THz wave technology is well known for its advantages in bioimaging applications. In fact, the imaging of THz metamaterial can also enhance the sensing resolution of the biosample. Lee et al. proposed a nano-slot metamaterial chip to image residual methomyl on an apple peel, differentiating the contaminated and clean areas [66]. The same research group later developed a similar nano-slot metamaterial for imaging a mouse cerebellum and a fingerprint [67], as shown in Figure 2d. Without the metamaterial, the THz reflectivity of white matter in the cerebellum is only 2% higher than that of gray matter, which increases to 4% with the metamaterial. Metamaterial imaging was also applied to detect a fingerprint pattern with an average pitch of about 250 μm, which reached the THz wave diffraction limit. To further increase THz metamaterial imaging sensitivity, Jahani et al. proposed a microarray metamaterial integrated with a microfluidic system to realize the THz imaging system. A detection limit of 0.41 nanoparticle/µm^2^ was demonstrated, and a concentration of extracellular vesicles as low as 204 femtomolar was measured in real-time imaging [68].

## 3. Different Metamaterial Resonances

The enhanced detection sensitivity observed in THz metamaterials is primarily based on the strong optical resonances induced in the metamaterials. This section discusses and compares the sensitivity realized through different THz resonances in the metamaterials, including EIT-like resonance, Fano resonance, toroid resonance and SPP resonance.

### 3.1. EIT-like Resonances

**Figure 3:**
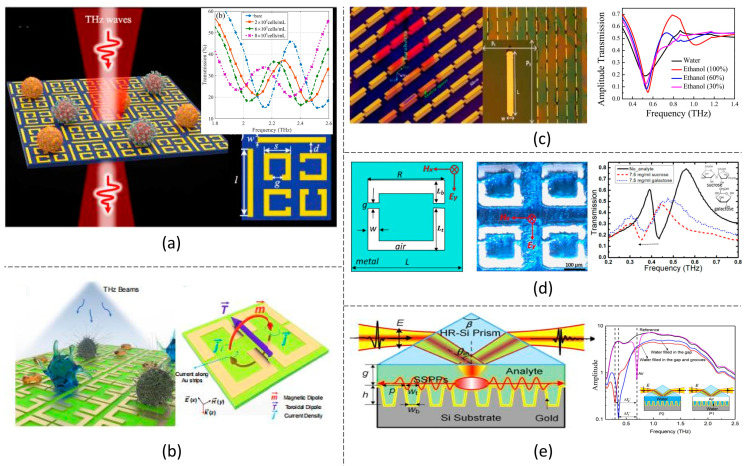
Biosensing based on different THz metamaterial resonances. (**a**) EIT-like resonance [71]; (**b**) toroidal resonance [79]; (**c**) Fano resonance in stacked nanorods [76]; (**d**) Fanon resonance on free-standing substrate [75]; and (**e**) surface plasmon polariton [83].

The EIT phenomenon is initially observed in a coherently driven atomic system, in which an ultra-narrow absorption dip is observed in the absorbance spectrum. This is due to the quantum interference effect between different atomic levels. The couplings among different energy levels in this phenomenon innovate the EIT-like metamaterials in which two different resonant modes are artificially designed [27], namely the radiative mode (bright mode) and the non-radiative mode (dark mode). In EIT-like metamaterials, the radiative mode is excited directly by the EM wave under certain incident conditions, resulting in a transmission peak (absorption dip) due to the energy dissipation. Due to the destructive interference induced between the radiative and non-radiative modes, the radiative loss is significantly suppressed at the non-radiative mode frequency. Thus, a narrow transmission band is obtained at this frequency. Because the transmission is due to the strong interference between the two modes, a slight environmental change will significantly affect the transmission peak frequency and magnitude, which can be applied for highly sensitive biosensing [69]. In a typical EIT-like metamaterial consisting of unit cells with a cut-wire structure and a split-ring resonator, the electrical dipole was induced in the cut wire, and the magnetic dipole was induced in the split ring when the incident polarization was along the cut-wire direction [70]. The radiative electrical dipole and the non-radiative magnetic dipole caused a transmission window with a peak at about 1.3 THz. The transmission peak was shifted when the metamaterial was covered with lung cancer cells and realized a detection of limit of 5 × 10^5^ cells/mL. Later, Zhang et al. proposed a polarization-independent EIT metamaterial, as shown in Figure 3a, which successfully differentiated the mutant and wild types of glioma cells [71] even though both cells were at different concentrations. This was achieved by investigating both the EIT resonant frequency shift and the transmission amplitude change.

### 3.2. Fano Resonances

Fano resonance is another metamaterial resonance for biosensing, which manifests itself as a sharp asymmetric line shape resonance induced from the coupling between a narrow discrete resonance and a broad spectral line. In metamaterials, the Fano resonance can be realized by breaking the symmetry of the meta-atoms. Fano resonances in both transmitted and reflected THz wave spectra have been reported for biosensing. For example, in a metamaterial with an asymmetric double-cut-wire unit cell, the transmitted Fano resonance achieved a 160 GHz/RIU frequency shift for the water–methanol mixture sensing [72]. A reflective Fano resonance was also reported in an asymmetric gap metamaterial, demonstrating a sensitivity of about 112 GHz/RIU for measuring the concentration of the ethanol solution covering the metamaterial [73]. To improve the Fano resonance sensitivity, a metamaterial with an ultra-thin substrate [74] and a metamaterial free of substrate [75] (Figure 3d) were proposed. The ultra-thin metamaterial realized a theoretical sensitivity of 240 GHz/RIU near the 1.13 THz transmission dip, and the free-standing metamaterial reached a theoretical sensitivity of 91.7 GHz/RIU with the initial Fano resonance transmission dip at 0.45 THz. To compare the sensitivity of the above metamaterials with different Fano resonance frequencies, we normalized the sensitivity by the initial resonant frequency. Considering the initial Fano resonant frequencies were at about 1.3 THz in [72], 1.55 THz in [73], 1.33 THz in [74] and 0.45 THz in [75], the normalized sensitivities were 0.12/RIU, 0.07/RIU, 0.21/RIU and 0.20/RIU, respectively. This indicates that a thin or free substrate can enhance sensitivity in the Fano metamaterial. Furthermore, a metamaterial with two-layer stacked nanorods was proposed, which induced Fano resonance due to the asymmetric current flow on the nanorods, as shown in Figure 3c [76]. An ultra-high sensitivity of 1 THz/RIU was claimed at the Fano transmission dip around 1 THz when confining the analyte as a 2 μm thick spacer between the two nanorod layers. This reaches a normalized sensitivity of 1/RIU, superior to the above works. However, it is necessary to point out that the confinement of the analyte in a 2 μm thick space is quite challenging for the sample fabrication in reality.

### 3.3. Toroidal Resonances

In recent years, toroidal resonance metamaterial has attracted much attention for biosensing research. In toroidal resonance, the surface current flows on a torus structure along its meridians, which leads to highly confined electrical and magnetic fields and induces strong light–matter interactions. As a result, the toroidal resonant metamaterial is a good candidate for detecting the biosample [77,78,79], which was demonstrated with even higher sensitivity than that of Fano resonances [80]. The toroidal metamaterial is usually designed with a pair of split-ring structures arranged symmetrically [79,80,81,82]. In work [79], as shown in Figure 3b, a toroidal metamaterial on a 500 μm thick quartz glass substrate achieved a refractive-index sensitivity of 485.3 GHz/RIU at around 2.4 THz, which is equal to a normalized sensitivity of 0.2 /RIU. This is comparable to the Fano resonance metamaterial with ultra-thin substrate [74] and free-standing metamaterial [75]. Therefore, the toroidal-resonance-based metamaterial could potentially realize ultra-high sensitivity for biosensing applications. Meanwhile, the toroidal metamaterial was demonstrated to be incident-angle-insensitive, offering more convenience in the measurement [79]. Iron is used as the magnetic resonator in the THz metamaterial to enhance further the toroidal resonance for biosensing, which enhanced the magnetic resonance and demonstrated a high *Q* factor ~18 for protein detection [77].

### 3.4. SPP Resonances

SPP stems from light coupling and the collective electron plasma oscillations at a metal–dielectric interface. It has been long and timely applied for high-sensitivity detection due to the strong wave confinement in a sub-wavelength scale. The excitation of SPP usually occurs in the ultraviolet or the visible region. The THz wave, however, is much less confined as the penetration depth into metal is much shorter than the corresponding wavelength. For enhancing the confinement of the THz wave, a metamaterial is designed to satisfy the momentum match condition of SPP, which results in a so-called spoof surface plasmon metamaterial [84]. A typical spoof surface plasmon metamaterial design consists of metal hole structures with thicknesses from several to hundreds of micrometers [85,86,87]. In the work [86], a sensitivity as high as 0.4 THz/RIU was obtained at the resonant frequency of about 0.3 THz for the sensing of ^12^CH_3_OH and its D isotope ^12^CD_3_OD, which gave an ultra-high normalized sensitivity of 1.3/RIU. Metamaterials consisting of a dielectric groove array coated with several-hundred-nanometers-thick metal were also reported for the spoof surface plasmon realization [83,88,89,90]. The THz wave is obliquely incident and coupled to the groove metamaterial through a prism or a blade to satisfy the phase-matching condition for the spoof surface plasmon, as shown in Figure 3e. The highly confined wave in the groove structure enhanced the sensitivity, and FOM up to 49 and 20 were claimed for lossless fluids and low-loss fluids detection, respectively [88], where FOM was defined as the change in resonance frequency per RIU divided by the width of the resonance. Later, a planar metamaterial composed of corrugated metallic stripes perforated by rectangular grooves was reported to realize the spoof SPR [91,92]. The planar spoof SPR metamaterial obtained a frequency sensitivity of 1.966 THz/RIU and FOM of 19.86 [92]. In addition, the spoof SPR allows the extraction of the complex refractive index of different viruses by simultaneously investigating the resonant frequency and absorption magnitude of the spoof surface plasmon [93,94]. This could provide more information on the analytes and improve the biosensing accuracy.

### 3.5. Sensing Based on Quasi-BIC

In recent years, metamaterials based on bound states in the continuum (BIC) were proposed in biosensing applications. BIC, which initially refers to a state in the continuum of the dispersion relation while it does not radiate to the far field in the quantum mechanics, could induce an infinite *Q* factor resonance theoretically. For the realistic application, a quasi-BIC with tailorable *Q* factor resonance was developed based on different mechanisms to improve the metamaterial sensing performance [95]. A quasi-BIC was reported in the metamaterial by inducing interference between a leaky electric dipole resonance and a bound toroidal dipole mode, which is symmetry-protected. Based on this metamaterial, interleukin-6 with concentration of 1 nM was detected, and a resonant frequency shift of 23 GHz was observed [96]. By breaking the metamaterial symmetry, the quasi-BIC can also be generated for ultra-high-sensitive refractive-index sensing [97,98]. For example, in [98], a refractive-index sensitivity of 170.58 GHz/RIU was obtained in an all-dielectric column-structured THz metamaterial with broken symmetry that supports quasi-BIC. A germanium film as thin as 7 nm was also sensed on the THz BIC metasurface, which corresponded to a deep subwavelength length scale [99]. Very recently, a magnetic dipole quasi-BIC was reported in a split-ring resonator consisting of THz metamaterial, which detected an ultra-low concentration of C-reactive protein down to 1 pM with sensitivity of 674 GHz/RIU [100].

## 4. Non-Conventional Materials

Conventionally, THz metamaterials are based on metallic unit cells on a solid dielectric substrate to realize the desired THz resonance discussed above. Recently, non-conventional materials such as dielectric materials, one-dimensional and two-dimensional materials were proposed for metamaterials to improve the sensing performance in different aspects such as the working frequency band and sensing linearity. In this section, we will discuss the improved biosensing performance of the metamaterial consisting of non-conventional materials.

### 4.1. All-Dielectric Metamaterials

**Figure 4:**
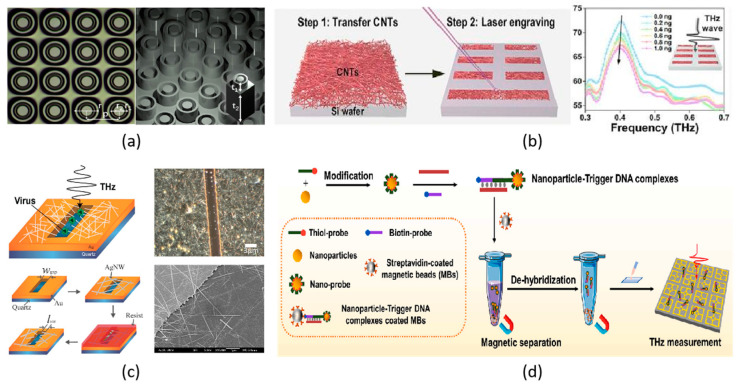
Biosensing based on different materials. (**a**) All-dielectric metamaterial [101]; (**b**) carbon-nanotube-based metamaterial [108]; (**c**) AgNWs-assisted metamaterial [109]; and (**d**) AgNPs-assisted metamaterial [110].

Metamaterials with metal unit cells usually suffer from high ohmic loss and narrow resonant bandwidth. A complex meta-atom structure has to be developed to increase the resonant band. As an alternative, doped silicon (Si) material was proposed to construct the unit cell, which is compatible with the CMOS process and can realize low ohmic loss and broad resonant bandwidth with a simple meta-atom structure [101,102]. The Si-based THz metamaterials also exhibit high stability to change in temperature and humility, making them suitable for longitudinal-based biosensing. In addition, the resonance can be dynamically tuned through optical pumping, which changes the free carrier density in Si and can optimize the sensitivity [101,103]. For example, in a doped Si-based metamaterial with a unit cell structure consisting of a ring and a cylinder, as shown in Figure 4a, strong absorption resonances were induced under optical pumping [103]. The chlorpyrifos down to 0.1 mgL^−1^ was detected, which changed the absorption amplitude and resonant frequency. A linearity of 0.9943 between the chlorpyrifos concentration and the absorption amplitude and 0.9750 between the chlorpyrifos concentration and the absorption frequency was observed. Similar good-concentration linearity was obtained in other Si-based THz metamaterials for other analyte testing, such as benomyl and tricyclazole solutions [104]. In addition, multi-band resonances, which usually require complex unit cell structures in metallic metamaterial, can be achieved through a simple-structured dielectric metamaterial. This simplified metamaterial design could perform unique advantages in bio-detection [103,105]. As reported in [103], quad-band and tri-band resonance were obtained in the absorption spectrum through a Si-based metamaterial consisting of grating structured unit cells with different grating depths, which was applied for chlorpyrifos solution detection with more sensing freedom. On the other hand, the angle scanning spectrum of the dielectric THz metamaterial was proposed for the detection of saccharides, such as lactose and glucose, with a detection limit of 1.53 mg cm^−2^ and 1.54 mg cm^−2^ [106], and acids, such as tyrosine and santonin, with a detection limit of 6.7 mg cm^−2^ and 59.35 mg cm^−2^, respectively [107].

### 4.2. Carbon-Assisted Metamaterials

Graphene, a two-dimensional carbon nanomaterial, has attracted much attention in various research works for its impressive intrinsic electronic, thermal, mechanical and optical properties. By integrating a graphene layer into a THz metamaterial, the resonance in the metamaterial could be tuned by controlling the Fermi level of the graphene under electrical bias [90,111,112]. As a result, biosensing for a fixed metamaterial can be optimized dynamically. The graphene interacts with molecules with π-electrons through the π−π stacking effect., realizing a THz metamaterial with selective biosensing [113]. The metamaterial exhibited a relative reflectance change above 30% when detecting 1 mg/L chlorpyrifos-methyl with π-electrons, while comparably, this value is only around 15% when detecting fructose without π-electrons. On the other hand, highly doped graphene becomes a plasmonic material to interact with the THz wave and induce collective oscillations of valence electrons [114]. Therefore, graphene can replace metal as the active element in the THz metamaterial for biosensing by generating proper resonance, such as absorption peak or EIT resonance, as discussed in the earlier section, which brings more tunable sensitivity and flexibility for the biosensor. Another carbon nanomaterial that attracts much research attention is the one-dimensional carbon nanotube (CNT) [115], which has been proven to have high sensitivity for the selective sensing of dopamine [116]. Furthermore, CNTs can strongly interact with π-conjugated molecules via π−π stacking as graphene does and adsorb analytes such as saccharides via the van der Waals force. Based on this principle, a CNT-assisted THz metamaterial biosensor was designed, as shown in Figure 4b, to have a high detection limit of 40 ng/mL and 30 ng/mL for lactose sensing and glucose sensing, respectively [108].

### 4.3. NW and NP Metamaterials

AuNPs usually exhibit surface plasmon resonance in the visible-light range and are applied for biosensing applications, which is rarely found in the THz regime. Instead, AuNPs were demonstrated to improve the sensitivity of THz metamaterials for their high refractive index compared to the bio-analytes [110,117]. This also explains why AuNPs are favored over AgNPs, as AuNPs have a higher refractive index in the THz regime. AuNPs can be conjugated with bio-analytes, such as avidin, through electrostatic force [118]. When coated on the metamaterial, it manifests more than 1000-fold sensitivity improvement for the avidin detection with a detection limit as low as 7.8 fmol and a good sensing linearity of 0.9371. Ultra-highly sensitive detections for other types of protein were also demonstrated in which the protein is conjugated with AuNPs through antibody binding [81,119]. Usually, AuNPs with a larger diameter induce higher sensitivity [119], which has an upper limitation due to the packing of AuNPs on the metamaterial structure, as shown in Figure 4d [110]. AuNPs will also assist in detecting nucleic acids, the detection process of which will be discussed in detail in Section 5.2. We compared the sensing performance of THz metasurfaces based on the above mechanisms as shown in Table 1.

## 5. THz Metamaterial Biosensors Targeting Different Analytes

This section summarized the THz metamaterial biosensing for different analyte categories. It has been demonstrated that THz metamaterial performs high-sensitivity detection for various analytes, including chemical reagents, biomolecules, microorganisms and cells.

### 5.1. Chemical Reagents

Chemical-reagent sensing is essential in food safety, environmental monitoring, drug development and industrial control. THz metamaterials have been reported to detect various chemical reagents such as alcohols [63,73,85,86,120,121], sugars [57,87,122,123,124], acid [125] and biotin [56,87], etc. The sensing ability is usually characterized by the detection limit, sensitivity and figure of merit. The sensitivity is the resonant frequency shift per RIU change, and the figure of merit is the sensitivity divided by the full-width half-maximum value. For example, sugar detection reported a detection limit of 9 mg/dL for glucose sensing [122] and 20 mg/dL for lactose sensing [123]. The types of chemical reagents can also be identified by measuring the THz spectrum of the metamaterials, which has been reported to distinguish different alcohol isotopes [86] or other types of fatty acids [125]. In the alcohol isotope detection, the ^12^CH_3_OH and ^12^CD_3_OD with different elements, H and D, induced a surface plasma resonance shift of 0.02 THz between each other through the enhanced THz metamaterial. For different fatty-acid detection, a 0.1 THz resonance shift was observed in the metamaterial transmission spectrum due to various chemical bonds in the acids, which was not observable in Raman spectra technology.

Another important application of THz metamaterials in chemical analysis is pesticide detection [101,103,104,113,126,127,128]. Among different pesticides, chlorpyrifos detection received high attention for its wide use in different areas, including crops, animals and living environments. Despite its effectiveness in killing pests like insects and worms, the residual chlorpyrifos will affect human health even at a minute trace amount. Xu et al. designed a metal-pad-structured THz metamaterial, which realized a detection limit of 0.204 mg/L for chlorpyrifos sensing [126]. The same group also detected a trace amount of chlorpyrifos-methyl down to 0.2 ng with the graphene-assisted THz metamaterial [113]. The above detection relies on the THz resonance shift, which is time-saving and cost-effective, having apparent advantages over chemical-sensing approaches.

### 5.2. Biomolecules

Nowadays, biomolecules are widely used in biology, the agriculture industry, health care and drug development, medicine, etc. Some biomolecules are also used as an essential indicator for diagnosing diseases such as cancer. Therefore, detecting the type and concentration of biomolecules is crucial in the above areas. THz-metamaterial-assisted biomolecule sensing has been studied widely for proteins, nuclear acid and insulin detection. BSA is a small, stable, moderately non-reactive protein. It has been applied in various biomedical areas such as immunohistochemistry and modeling for other serum albumin proteins, especially human serum albumin, due to being up to 76% structurally homologous to human serum albumin [12]. Metamaterials with different unit cell structures, such as split rings [48], bow ties [120], thin slabs [129] and hollow-dumbbell patterns [130], were proposed to improve the sensitivity of BSA detection. The experiment results showed that the BSA would induce a 72.81 GHz/(ng/mm^2^) redshift of metamaterial resonance, and a detection limit of 0.035 mg/mL was realized [130]. Insulin, a major indicator of type II diabetes, was also sensed by the THz metamaterial for concentration analysis [131]. High concentration sensitivity at 85 GHz/RIU and a detection limit of insulin at about 0.05 IU/μL were achieved through a U and H hybrid-shaped metamaterial, where IU is the insulin unit.

Metamaterials are also applied to detect antigens, such as cancer markers, with great potential for label-free and rapid specificity detection [132,133,134]. The metamaterial is usually bound with a capture antibody to achieve high specificity. The binding of a specific antigen to the antibody increases the contact area between the antigen and metamaterial, which improves the antigen sensitivity. Based on this method, Geng et al. proposed a metamaterial with split-ring resonator unit cells and detected the alpha-fetoprotein at a low concentration of 25.24 ng /mL [135]. In addition, Cui et al. designed another metamaterial with double split-ring resonator unit cells, which successfully detected 500 ng/mL of carcinoembryonic antigen. The detection limit in both cases is superior to the clinical standards, indicating a promising sensing method [136].

**Figure 5:**
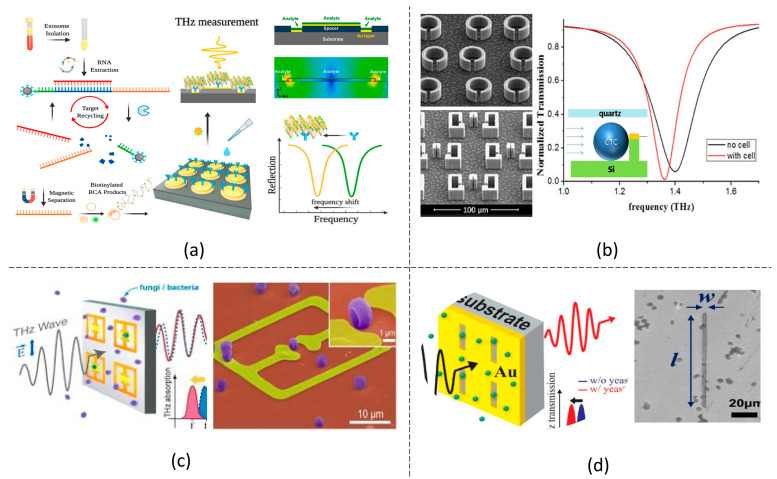
Biosensing for different bio-analytes of (**a**) mRNA [137], (**b**) CTCs [147], (**c**) fungi/bacterial [140], and (**d**) yeast [141].

Detecting nuclear acid also attracts much attention for its applications in medical diagnosis, pharmaceutics and mutagenesis applications. The ribonucleic acid (RNA) to be detected is usually amplified through processes such as rolling-circle amplification [137] and strand displacement amplification [110]. For example, the micro-RNA was amplified and then captured by a THz metamaterial, as shown in Figure 5a. This resulted in a redshift of the resonant peak, achieving a detection limit down to 84 aM [137]. Sensitive deoxyribonucleic acid (DNA) detection was also demonstrated in the THz metamaterial, which successfully identified a transgenic tomato genome [138]. Furthermore, graphene-assisted metamaterials improved the detection sensitivity for single-stranded DNAs at the nano-mole level [139] and successfully detected DNA short sequences of 100 nM [54].

### 5.3. Microorganisms and Cells

Detection and identification of viruses and bacteria are of intense interest for preventing infectious disease outbreaks and diagnosing infections such as sepsis. An on-site platform is highly desired for microorganism detection, which can be fulfilled with THz metamaterials [51,93]. For example, THz metamaterials with split-ring resonator unit cells [140] and slot pattern unit cells [141], as shown in Figure 5d, were demonstrated for high-speed and on-site detection of *Escherichia coli* (*E. coli*), penicillia and yeast, which can be fulfilled either in the ambient or aqueous conditions. The sizes of the above microorganisms are on the same scale as the gaps in the meta-atom. Therefore, even with an ultra-small amount of microorganisms, it could induce a significant resonance change in the metamaterial. It was demonstrated that a surface density of 0.113/μm^2^ can be detected for the penicillia, 0.028/μm^2^ for the yeast cells and 0.090/μm^2^ for *E. coli*, respectively, as shown in Figure 5c [140]. The sensitivity can be improved by introducing nanometer-sized structures in the THz metamaterial. For example, Park et al. reported an on-site detection of the PRD1 virus with a THz metamaterial with split-ring resonator unit cells. The sensitivity, evaluated by the transmission dip shift per viral particle per unit area, was significantly increased from 6 GHz·μm^2^/particle to 80 GHz·μm^2^/particle by simply decreasing the split width of the unit cell from 3 μm to 200 nm [142]. Another THz metamaterial with micrometer-sized slot unit cells initially exhibited a sensitivity of 12.8 GHz·μm^2^/particle for the PRD1 virus detection. The sensitivity has been reported to increase to 32.7 GHz·μm^2^/particle after depositing AgNWs on the metamaterial, as shown in Figure 4c [109].

Detection of cancer cells or tumor cells is essential to diagnose different cancer diseases. THz-metamaterial-based biosensors detect cells through transmitted or absorbed spectrum change, which is highly sensitive, non-destructive and time-saving, and has been applied for the detection or identification of oral cancer cells [69,143,144], lung cancer cells [70,79], breast cells [145] and other tumor cells [71,146]. For example, an EIT-like metamaterial manifested a transmission resonance shift from 24 GHz to 50 GHz when the cultured lung cancer cell A549 concentration increased from 0.5 × 10^5^ to 5 × 10^5^ cells/mL [70]. The spectrum shift can also be utilized to monitor the anti-cancer drug effect on the cancer cell, which could benefit new drug discovery and cancer diagnosis. It was demonstrated that the resonance of the THz metamaterial would shift at different frequencies when the oral cancer cell is cultured on the metamaterial and treated with varying concentrations of the drug for different durations [69]. This will be useful for controlling drug treatment on the cancer diagnosis. In addition, the metamaterial can also be designed to trap circulating tumor cells (CTCs), which are rare in blood samples and hard to detect. An enrichment of CTC was realized through the metamaterial due to the mechanical trap, as shown in Figure 5b, and a 38 GHz resonant frequency was achieved [147].

## 6. Conclusions and Future Perspectives

Biosensing based on THz metamaterials holds tremendous potential for various applications in medicine, agriculture and environmental monitoring. One of the significant advantages of biosensing with THz metamaterials is their ability to provide label-free and non-invasive detection with high sensitivity. The transmission, absorption and imaging of the THz wave can be used for bio-detection, which offers increased flexibility and convenience. High sensitivity is usually achieved based on the THz resonance in the metamaterials, which could be tailored due to the artificial attribute of the metamaterials. Different resonances, such as EIT-resonance, Fano resonance, toroidal resonance, etc., have been used for high-sensitivity detection due to hot spots induced in the metamaterials. Enhancing the resonances through non-conventional materials such as graphene, CNT and AgNP will further improve THz metamaterial sensing performance. As THz waves can penetrate through various biomaterials invasively, the THz metamaterials enable the measurement of chemical and structural information in biological samples without needing sample pre-treatment. THz metamaterials can be designed to exhibit highly sensitive and selective responses to specific biological molecules, such as proteins, DNA or viruses. Functionalizing the metamaterial structures with specific receptors or antibodies can selectively bind them to target molecules and induce recognizable changes in the THz wave properties. These changes can then be measured and analyzed to identify and quantify the presence of specific biological substances. In addition, THz metamaterial biosensors offer the potential for real-time and label-free monitoring of dynamic biological processes. They can be employed for continuous longitudinal monitoring of biochemical reactions, enzymatic activities and cell behaviors. This real-time monitoring capability can significantly enhance our understanding of biological processes and facilitate the development of novel diagnostic tools and therapies.

However, challenges still need to be addressed for the practical implementation of THz metamaterial biosensing. These include improving the specificity and the reproducibility of the biosensor response, as well as reducing the complexity and cost of fabrication. The specificity of the biosensing is a problem when only one THz wave signal is used for the biosensing. For example, the resonant frequency shift of the THz metamaterial can be caused by either different analyte concentrations or different analyte types. Multi-signal detection methods could solve this problem by simultaneously measuring the change of multi-parameters, including resonant frequency, resonant amplitude, polarization rotation and multi-band resonance. Reproducibility also remains a challenge in THz metamaterial sensing. The noise from the environmental change of the sample on the metamaterial will cause the metamaterial signal to shift and affect the detection results. Therefore, a stable environment, such as a microfluidic chamber, can be used for the detection. Furthermore, integrating THz metamaterials with miniaturized and portable sensing platforms would be crucial to enable their widespread adoption in various settings. Overall, the outlook for biosensing through THz metamaterials is promising. Continued advancements in metamaterial design, fabrication techniques and integration approaches are expected to unlock new opportunities for sensitive, selective and real-time biosensing, revolutionizing fields such as health care, agriculture and environmental monitoring.

## Figures and Tables

**Table 1:** Sensing performance comparisons of THz metasurfaces based on different mechanisms.

Detection Basis		Metamaterial Structure	Analytes	Sensitivity	Resolution	Linearity	Ref
Sensing principle	Transmission dip shift	Split-ring resonator	BSA	95 GHz/(mmol/L)	17.7 µmol/L	-	[48]
		Mirror-asymmetric SRRs	A549 cells	213 GHz for 5 × 10^5^ cells/mL	-	-	[53]
	Absorption peak shift	Loop shape	Organochlorine pesticide	-	0.213 mg/L	0.9863	[55]
		Bi-layer structure	Biotin	153 GHz/µM	-	-	[56]
		Microchannel assisted	Ar/ethanol/glucose	0.2 THz/RIU	-	-	[57]
		Square and split-ring hybrid structure	2,4-dichlorophenoxyacetic acid	-	0.1 ppm	-	[58]
	Polarization rotation	Hexagon structure	Tumor cells	-	3 × 10^3^ cells/mL	-	[60]
	EIT-like resonance	Cut-wire structure and a split-ring resonator	lung cancer cells	-	5 × 10^5^ cells/mL	-	[70]
	Fano resonance	Asymmetric double-cut wire	water-methanol mixture	160 GHz/RIU	-	-	[72]
		Asymmetric gap	ethanol solution	112 GHz/RIU	-	-	[73]
		Ultra-thin substrate	protein A/G	240 GHz/RIU	-	-	[74]
		Free of substrate	galactose	91.7 GHz/RIU	-	-	[75]
		Stacked nanorods	IPA/metanol/ethanol	1 THz/RIU	-	-	[76]
	Toroidal resonance	Symmetrically arranged split-ring pair	Lung cancer cells	485.3 GHz/RIU	-	-	[79]
	SPP resonance	Metal holes	^12^ CH_3_OH and ^12^ CD_3_OD	0.4 THz/RIU	-	-	[86]
	Quasi-BIC	Triple micro-rods	Interleukin-6	-	1 nM	-	[96]
		Split-ring resonator	C-reactive protein	674 GHz/RIU	1 pM	-	[100]
Special materials	All-dielectric materials	Ring and a cylinder	Chlorpyrifos	-	0.1 mg/L	0.9943	[103]
		Cylinder dimers	Lactose	-	1.53 mg /cm^2^	-	[106]
			Glucose	-	1.54 mg /cm^2^	-	[106]
		Zigzag array of tilted silicon bars	Tyrosine	-	6.7 mg/cm^2^	-	[107]
			Santonin	-	59.35 mg/cm^2^	-	[107]
	Carbon assisted	CNT coated	Lactose	-	40 ng/mL	-	[108]
			Glucose	-	30 ng/ml	-	[108]
	NW and NP	AuNPs conjugated	Avidin	-	7.8 fmol	0.9371	[118]
